# Supplementary material for: Relationship between Composite Dietary Antioxidant Index and Aging
Source: Healthcare (Basel). 2023 Oct 12;11(20):2722. doi: 10.3390/healthcare11202722 (PMC10606125; doi:10.3390/healthcare11202722)
Supplement: Supplementary file 1 [file healthcare-11-02722-s001.zip › healthcare-2543916-supplementary.pdf]

Supplementary method S1

These factors have been widely established to be associated with aging. David et al. demonstrated that smoking reduces average lifespan by 7 years, and tobacco consumption shortens healthy life expectancy by 14 years [1]. Polliana et al. found a close association between alcohol consumption and brain aging and cognitive impairments[2]. Furthermore, comorbidities such as hypertension, diabetes, and coronary heart disease are considered accompanying symptoms of aging. Regarding social characteristics, income, health insurance, and educational level have also been linked to individual aging[3-4].

[1] Bernhard D, Moser C, Backovic A, Wick G. Cigarette smoke--an aging accelerator? Exp Gerontol. 2007 Mar;42(3):160-5. doi: 10.1016/j.exger.2006.09.016.

[2] Nunes PT, Kipp BT, Reitz NL, Savage LM. Aging with alcohol-related brain damage: Critical brain circuits associated with cognitive dysfunction. Int Rev Neurobiol. 2019;148:101-168. doi: 10.1016/bs.irn.2019.09.002.

[3] Pothisiri W, Prasitsiriphon O, Aekplakorn W. Extent of aging across education and income subgroups in Thailand: Application of a characteristic-based age approach. PLoS One. 2020 Dec 8;15(12):e0243081. doi: 10.1371/journal.pone.0243081.

[4] Parker SW, Saenz J, Wong R. Health Insurance and the Aging: Evidence From the Seguro Popular Program in Mexico. Demography. 2018 Feb;55(1):361-386. doi: 10.1007/s13524-017-0645-4.

Figure S1. Flow chart.

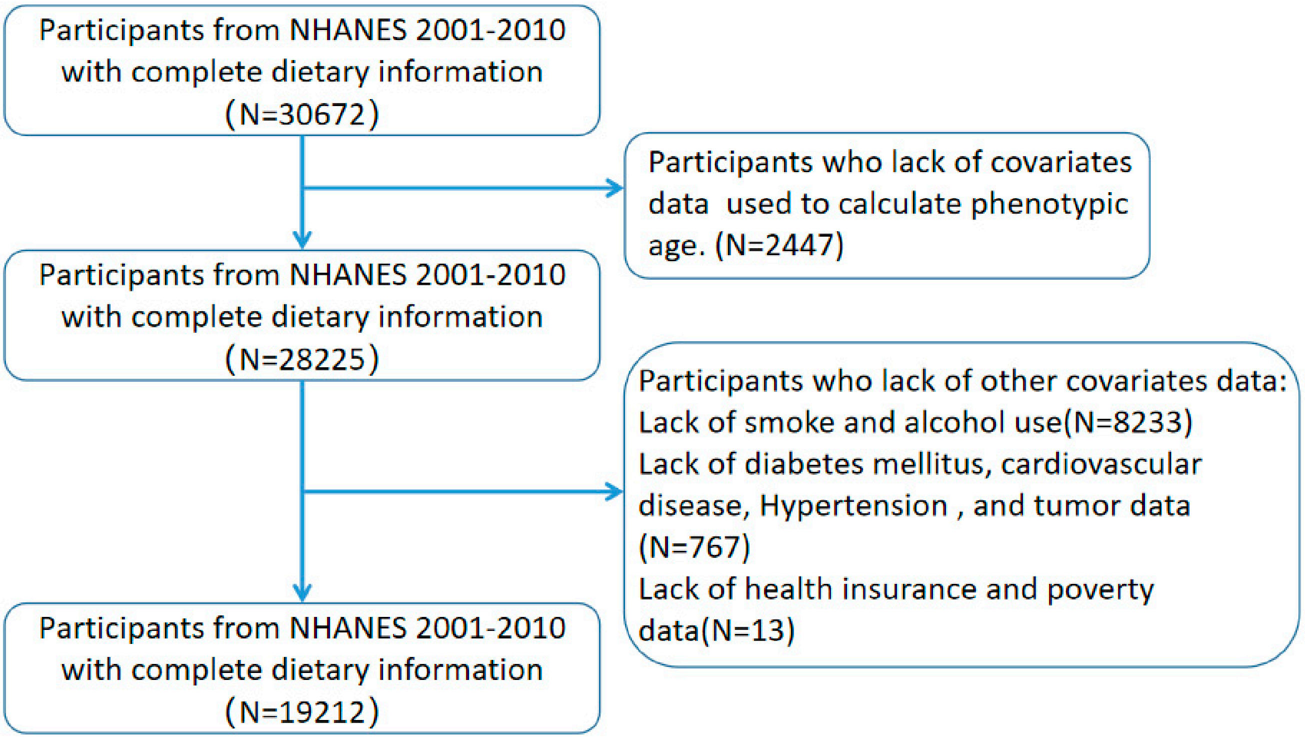

Figure S2. The correlation between CDAI and phenotypic age.

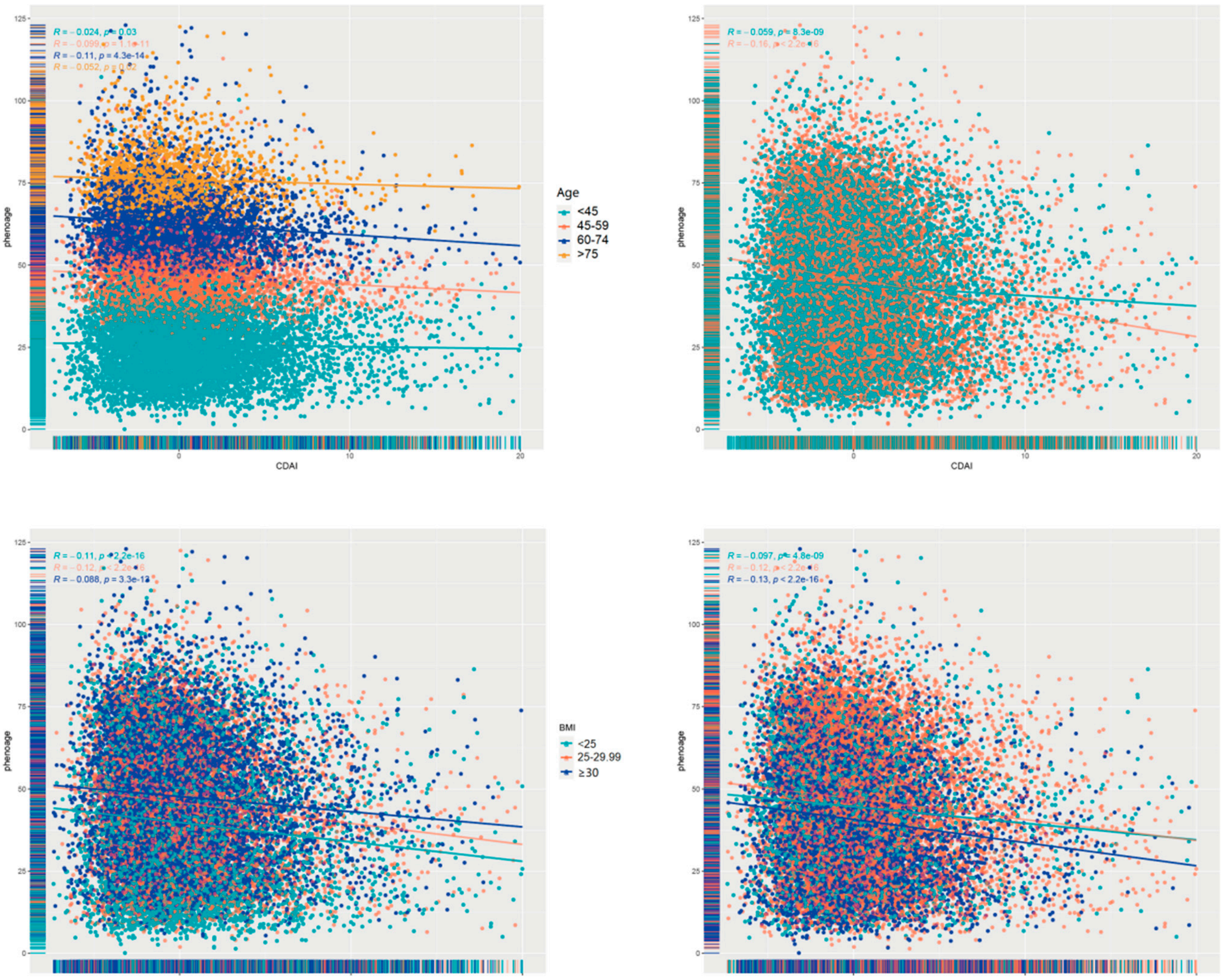

Figure S3. RCS of CDAI components and aging risk.

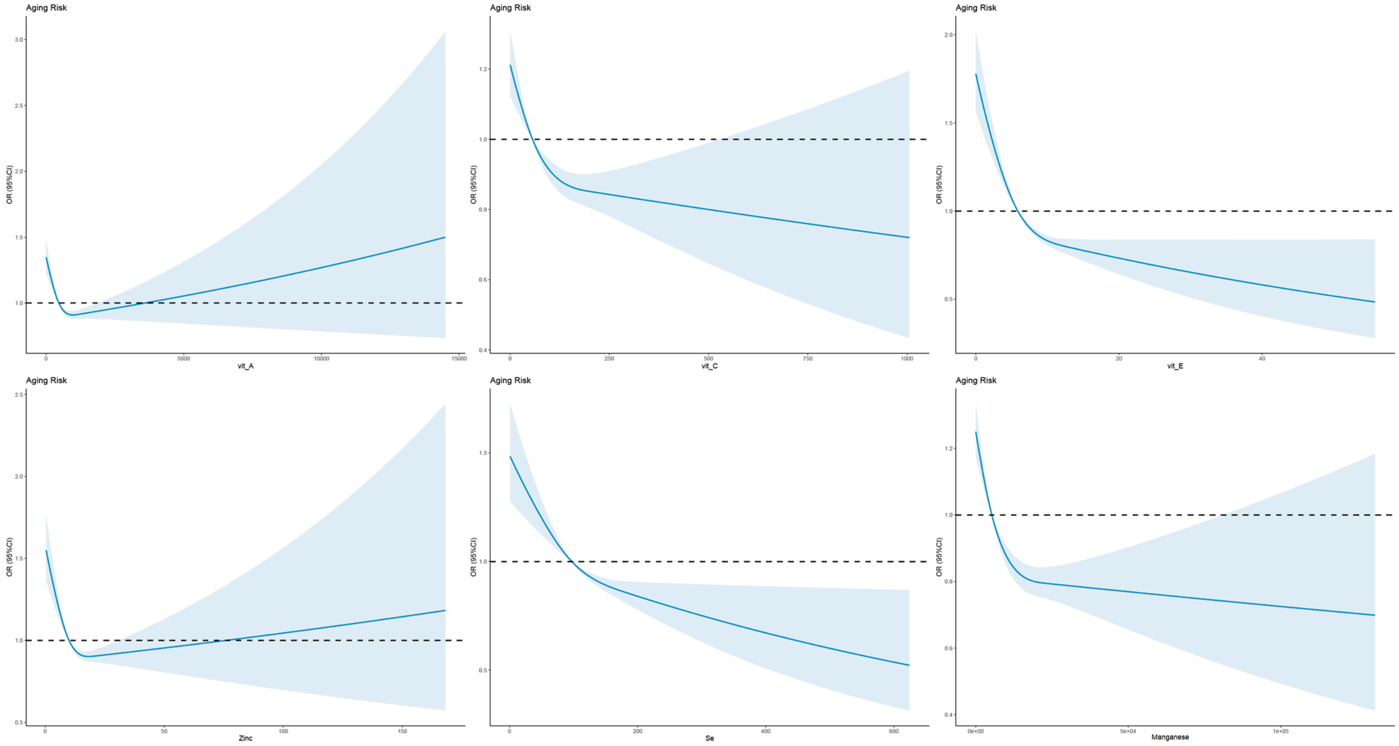

Notes: Model was adjusted for sex, race, BMI, education level, PIR, health insurance, smoke, alcohol, DM, CVD, hypertension, cancer history, CRP and albumin.

Table S1. The relationship between CDAI components and aging risk.

	OR(95%CI)*	p
vitamin A (Per SD)	1 (0.92,1.1)	0.969
vitamin C (Per SD)	0.94 (0.89,0.99)	0.019
vitamin E (Per SD)	0.86 (0.80,0.92)	<0.001
Zinc (Per SD)	0.95 (0.85,1.05)	0.300
Se (Per SD)	0.89 (0.83,0.96)	0.002
Manganese(Per SD)	0.89 (0.84,0.94)	<0.001

* Model was adjusted for sex, race, BMI, education level, PIR, health insurance, smoke, alcohol, DM, CVD, hypertension, cancer history, CRP and albumin.

Table S2. Sensitivity analysis.

	Excluding tumor patients (n=17428)		Excluding participants aged 75 and above (n=17241)		Include only participants from 2001 to 2006 (n=10000)	
CDAI	OR(95%CI)*	p	OR(95%CI)*	p	OR(95%CI)*	p
Per SD	0.91(0.85,0.96)	0.002	0.89(0.84,0.96)	0.001	0.92(0.83,1)	0.053
Low	Reference		Reference		Reference	
High	0.81(0.72,0.92)	0.001	0.80(0.71,0.91)	0.001	0.83(0.68,0.96)	0.037
Quantiles						
Q1	Reference		Reference		Reference	
Q2	0.92(0.76,1.12)	0.406	0.92(0.77,1.11)	0.380	0.89(0.74,1.07)	0.205
Q3	0.88(0.69,1.11)	0.279	0.89(0.71,1.11)	0.291	0.82(0.63,1.07)	0.135
Q4	0.84(0.70,0.96)	<0.001	0.82(0.69,0.98)	0.033	0.81(0.60,0.99)	0.045
Q5	0.69(0.56,0.84)	<0.001	0.67(0.55,0.81)	<0.001	0.74(0.56,0.96)	0.032
P for trend		0.002		0.002		0.028

* Model was adjusted for sex, race, BMI, education level, PIR, health insurance, smoke, alcohol, DM, CVD, hypertension, cancer history, CRP and albumin.
